# Unsupervised Machine Learning Approaches Reveal Distinct Phenotypes of Perceived Bladder Pain: A Pilot Study

**DOI:** 10.3389/fpain.2021.757878

**Published:** 2021-11-05

**Authors:** Patricia J. Mwesigwa, Nicholas J. Jackson, Ashley T. Caron, Falisha Kanji, James E. Ackerman, Jessica R. Webb, Victoria C. S. Scott, Karyn S. Eilber, David M. Underhill, Jennifer T. Anger, A. Lenore Ackerman

**Affiliations:** ^1^Department of Obstetrics and Gynecology, Center for Women's Pelvic Health, David Geffen School of Medicine at UCLA, Los Angeles, CA, United States; ^2^Department of Medicine, David Geffen School of Medicine at UCLA, Los Angeles, CA, United States; ^3^Division of Urology, Department of Surgery, Cedars-Sinai Medical Center, Los Angeles, CA, United States; ^4^Cedars-Sinai Medical Group, Department of Internal Medicine, Los Angeles, CA, United States; ^5^Department of Biomedical Sciences, Cedars-Sinai Medical Center, Los Angeles, CA, United States; ^6^Division of Pelvic Medicine and Reconstructive Surgery, Department of Urology, David Geffen School of Medicine at UCLA, Los Angeles, CA, United States

**Keywords:** interstitial cystitis, bladder pain syndrome, urinary symptoms, phenotypes, pelvic pain/discomfort, lower urinary tract symptoms, unsupervised machine learning

## Abstract

Interstitial cystitis/bladder pain syndrome (IC/BPS) is defined as an unpleasant sensation perceived to be related to the bladder with associated urinary symptoms. Due to difficulties discriminating pelvic visceral sensation, IC/BPS likely represents multiple phenotypes with different etiologies that present with overlapping symptomatic manifestations, which complicates clinical management. We hypothesized that unique bladder pain phenotypes or “symptomatic clusters” would be identifiable using machine learning analysis (unsupervised clustering) of validated patient-reported urinary and pain measures. Patients (*n* = 145) with pelvic pain/discomfort perceived to originate in the bladder and lower urinary tract symptoms answered validated questionnaires [OAB Questionnaire (OAB-q), O'Leary-Sant Indices (ICSI/ICPI), female Genitourinary Pain Index (fGUPI), and Pelvic Floor Disability Index (PFDI)]. In comparison to asymptomatic controls (*n* = 69), machine learning revealed three bladder pain phenotypes with unique, salient features. The first group chiefly describes urinary frequency and pain with the voiding cycle, in which bladder filling causes pain relieved by bladder emptying. The second group has fluctuating pelvic discomfort and straining to void, urinary frequency and urgency without incontinence, and a sensation of incomplete emptying without urinary retention. Pain in the third group was not associated with voiding, instead being more constant and focused on the urethra and vagina. While not utilized as a feature for clustering, subjects in the second and third groups were significantly younger than subjects in the first group and controls without pain. These phenotypes defined more homogeneous patient subgroups which responded to different therapies on chart review. Current approaches to the management of heterogenous populations of bladder pain patients are often ineffective, discouraging both patients and providers. The granularity of individual phenotypes provided by unsupervised clustering approaches can be exploited to help objectively define more homogeneous patient subgroups. Better differentiation of unique phenotypes within the larger group of pelvic pain patients is needed to move toward improvements in care and a better understanding of the etiologies of these painful symptoms.

## Introduction

Interstitial cystitis/bladder pain syndrome (IC/BPS) is a frequently debilitating, chronic condition whose central diagnostic feature is pain attributed to the bladder (1). Direct costs of care are high; IC/BPS is chronic, incurable, and frequently treatment-resistant. Estimates fluctuate widely, but community-based surveys suggest that as many as 7% of women in the US may express this symptom (2). However, IC/BPS is a diagnosis of exclusion, defined by the AUA/SUFU guidelines only “in the absence of infection or other identifiable causes” (3, 4). There is no international consensus on this definition or diagnostic criteria, which has made estimates of prevalence, treatment responses, mechanistic data, and long-term outcomes inconsistent and unreliable. As a result, many women with bladder pain symptoms may never receive an accurate diagnosis (2). A lack of diagnostic and prognostic indicators makes it challenging to assign effective care and identify appropriate therapies, leaving patients highly debilitated (5).

While the central feature of IC/BPS is the perception of bladder pain, patients exhibit a diverse range of accompanying genitourinary (GU) symptoms such as urinary urgency, frequency, nocturia, dyspareunia (sexual pain), pelvic pain, and incontinence (6). These types of symptoms overlap considerably with other GU tract pathologies such as overactive bladder (OAB), vaginitis, dysfunctional voiding, and other pelvic pain syndromes (7, 8). Early data from the NIH Multidisciplinary Approach to the Study of Chronic Pelvic Pain (MAPP) Research Network previously created separate measures of pain and urinary severity (9), observing that the severity of each varied independently. In addition, each symptom domain was associated with different, co-morbid features, such as depression, suggesting that the variations in urinary symptoms and other pain features may reflect fundamentally distinct disease subsets.

Despite this large symptomatic diversity, IC/BPS is typically managed and studied as a single clinical condition. The combination of inconsistent mechanistic data, the large diversity of symptom expression, and the inability of any specific treatment to prove effective in more than a subset of patients suggest that IC/BPS is not a single clinical condition, but instead may reflect several unique pathologies that manifest with similar, overlapping symptoms (10, 11). It is likely this heterogeneity in IC/BPS populations that has made scientific advances difficult, confounding potential insights into disease physiology. That lack of understanding has in turn limited progress in diagnosis, prevention, and treatment. We hypothesize that the current definition of IC/BPS, requiring only the perception of bladder discomfort and co-existing urinary symptoms, encompasses multiple, mechanistically distinct disease phenotypes. Progress in clinical care and management of IC/BPS requires refinement of our diagnostic and prognostic schema by identifying independent biologies or data that can be used to define each phenotype more objectively to correlate with pathogenesis and treatment outcomes. In this study, we define unique IC/BPS phenotypes through deeper clinical profiling and begin to examine the response of these newly defined phenotypes to different treatment modalities.

## Materials and Methods

### Patient Recruitment

After approval from the local Institutional Review Board (IRB# Pro00046154), 521 sequential patients complaining of any element of pelvic pain were evaluated from the Cedars-Sinai Women's Urology Clinic for possible inclusion. For the IC/BPS cohort (*n* = 145), we enrolled premenopausal women (maximum age of 45) suspected of having IC/BPS by their treating provider at initial consultation. Diagnosis was made by one of four board-certified, Female Pelvic Medicine and Reconstructive Surgery specialty urologists from a detailed history and physical exam using the definition established by the American Urological Association (AUA)/Society of Urodynamics, Female Pelvic Medicine and Urogenital Reconstruction (SUFU): IC/BPS is “an unpleasant sensation (pain, pressure, discomfort) perceived to be related to the urinary bladder, associated with lower urinary tract symptoms of more than 6 weeks duration, in the absence of infection or other identifiable causes” (12). For inclusion, patients could not have any other urologic diagnoses considered to explain their urinary or pain symptoms (such as overactive bladder, recurrent urinary tract infections, urinary retention, or dysfunctional voiding). To ensure the perception of bladder pain was a symptomatic feature in the IC/BPS cohort, we implemented an additional inclusion criterion requiring direct patient endorsement of bladder pain. Previously, in a retrospective comparison of self–reported symptoms for subjects with a range of GU symptoms, we created a novel measure of perceived bladder pain that accurately distinguishes IC/BPS from OAB and asymptomatic controls, known as the bladder pain composite index (BPCI) (7). To ensure a more homogeneous group specifically expressing pain attributable to the bladder, we used the criterion of a BPCI score >4 for inclusion. Asymptomatic, age-matched controls (*n* = 69) were recruited independently from patients seen in the same clinic who had been referred for urologic evaluation for a range of benign, asymptomatic findings, such as renal cyst or microscopic hematuria. All of these subjects had a BPCI score <3 and asymptomatic responses on all additional questionnaires.

Subjects who had previously undergone invasive therapies prior to evaluation for inclusion, such as bladder instillations, prior pelvic surgery, intradetrusor Botox®, or sacral neuromodulation, were excluded, as were patients with active urinary tract infection, pregnancy, diabetes, neurologic or rheumatic disease (e.g., multiple sclerosis), current smoking, or a vaginal pessary. As worsening pain in association with menstrual cycle can be a common feature of IC/BPS, subjects who experienced flares or worsening pain during phases of the menstrual cycle were allowed to participate, but were excluded if they complained only of isolated cyclic pain at menses (dysmenorrhea/menorrhalgia). Baseline demographics and clinical data including age, body mass index (BMI), comorbidities, past surgeries, and medication usage, including hormonal medications, were captured at enrollment.

### Symptomatic Assessment Instruments

After signing informed consent, subjects completed the female Genitourinary Pain Index (fGUPI) (13) and Interstitial Cystitis Symptom and Problems Indices (ICSI/ICPI), (14), to quantitate and describe the typical pelvic symptoms associated with bladder pain. The Overactive Bladder Questionnaire Short Form (OABq-SF), (15), and Pelvic Floor Distress Index short form (PFDI-20) (16)) were also administered to measure the nature, severity, and impact of other urinary and pelvic symptoms.

### Cluster Analysis

Cluster analysis methodology utilized a two-step approach to generate symptomatic clusters for the IC/BPS population. Controls were not included in the derivation of clusters. First, hierarchical clustering using Ward's method generated a cluster dendrogram (**Figure 2A**). This provided an estimation of the number of likely clusters within the studied population of IC/BPS subjects, of which two or three groups appeared the most appropriate. The elbow method, in which the explained variation in the data is plotted as a function of the number of possible clusters (14), implicated a cluster number of three as the optimal solution for the number of phenotypic groups in our cohort (**Figure 2B**). Second, we applied the *K*-means machine learning algorithm (17) as the principal clustering technique to divide the symptomatic bladder pain cohort into three (the *k* determined in step 1) subgroups using phenotypic variables derived from the symptomatic assessment instruments. All measurements were standardized using z scores for continuous variables and 0 or 1 for categorical variables. Continuous variables were log transformed to approximate a normal distribution where indicated. Patient cluster distribution visualized by principal coordinate analysis with the Bray-Curtis dissimilarity measure.

### Self-Organizing Maps

Self-organizing maps (SOM), an unsupervised technique of clustering and dimensionality reduction, consist of an arbitrary number of nodes where each node represents a point in the original, multi-dimensional input space. As new points are added, they are classified by pairwise Euclidean distance with the nearest neighboring node. The grid of nodes is trained such that nearby nodes resemble each other more than nodes that are further away. A final step uses hierarchical clustering to group the nodes into a user-defined number of metaclusters, which is then visualized in two-dimensional space (18). This analysis utilized a Gaussian neighborhood function, in which all nodes are adjusted in n-dimensional space toward the current data point, but closer nodes are displaced more. This contrasts with a circular function, in which only the nodes nearest to the closest node are adjusted and those adjustments are all of equal magnitude. Neighborhood relations (typically rectangular or hexagonal) dictate the topology (interconnectivity) of the SOM. We selected a rectangular topology, which has fewer connections between nodes. SOM size was 6 × 4 with 1,000,000 iterations at a learning rate of 0.1, which was determined experimentally to provide the greatest convergence (19). This solution resulted in a convergence index of 0.78.

### Cluster Stability

To ensure cluster assignment stability, we resampled a large number of replications (10,000) with replacement (bootstrapping) and identified the cluster assignments for each iteration. The Rand (20) and Jaccard (21) indices were used to assess the agreement between each reference clustering and the clustering obtained for the subsampled validation cohort; percentile bootstrap 95% confidence intervals were generated for the Rand and Jaccard indices. The Rand index represents the overall percent observed agreement in cluster assignment, while the Jaccard coefficient represents the percent overlap between cluster assignments. The Jaccard coefficient measures only the times when the same assignment was made as a proportion of the total times the cluster was assigned in either the original or bootstrap sample. As a result, the Jaccard coefficient is a more sensitive measure of cluster similarity by excluding instances where neither the original nor bootstrap sample assign an observation to the cluster. Jaccard coefficient values > 0.7 are generally considered to indicate very good agreement. A challenge with establishing agreement is that *K*-means randomly assigns observations to a new cluster given an arbitrary number designation; thus, an emergent cluster of the same observations may be assigned a different cluster number in the bootstrapped sample. To account for differences in cluster number assignment when determining these metrics, we reassigned cluster numbers based on the highest interrater reliability (kappa) between the original cluster and the bootstrapped clusters.

### Thematic Analysis

To determine if there were additional features common to each symptom cluster that could not be captured by the patient-reported outcomes administered in this study, we performed a modified thematic analysis approach. Descriptions of patient complaints and features were captured from the history of present illness and assessment of the specialist's initial consultation within the electronic medical record. Primary patient complaints/bothersome symptoms as well as anticipated symptomatic features (including aggravating and relieving factors, presence/absence and patterns of flares, most bothersome symptom, and terminology used to describe the nature of their bladder/pelvic pain) were cataloged by two reviewers blinded to both subject cluster assignment and the nature of the characteristic cluster symptomatic features.

### Treatment Responses

For the 145 IC/BPS subjects, treatment responses were assessed by chart review for therapies prescribed after inclusion. Response to treatment was noted to be positive if the immediate post-treatment note documented a positive patient perception of improvement with therapy (yes/no). Medical charts were reviewed by one of two researchers who were blinded to both subject cluster assignment and cluster features. While patients were categorized to specific clusters from data obtained prior to their treatments, there was no communication with the patients about this categorization or its hypotheses; patients chose their care plans and reported their responses according to the typical standard of care with their treating provider. Only those patients for whom a determination about responses to treatment could adequately be made were included in the analysis. Only 105 subjects could be included in this analysis, as we did not have adequate information to determine treatment responses for 40 subjects (27%), either because they were unable to complete the course of treatment or to follow-up with their treating provider after treatment due to clinic closures associated with the SARS-CoV2 pandemic. A proportion of the subjects within each phenotype responding to each treatment was determined (within group analysis) as was the proportion of total responders to each therapy for each phenotype (within cohort analysis).

### Statistical Analysis

The R Studio integrated development environment was used for the unsupervised clustering analysis with the stats (version 3.6.1), cluster (version 2.1.0), mclust (version 5.4.7) randomForest (version 4.6–14), kohonen (version 3.0.10), and popsom (version 5.2) packages. Differences in patients' demographic and clinical characteristics were compared by using the Wilcoxon signed rank tests for paired data and the Pearson chi square, Fisher exact, or Mann-Whitney *U*-tests for independent data as appropriate (2-tailed). Differences in proportions were compared using the two-sample *z*-test. Results are considered significant at an alpha level <0.05.

## Results

### Objective Symptom Measure in Combination With Physician Diagnosis Provides Better Identification of IC/BPS Subjects Than Clinician Diagnosis Alone

**Figure 1:**
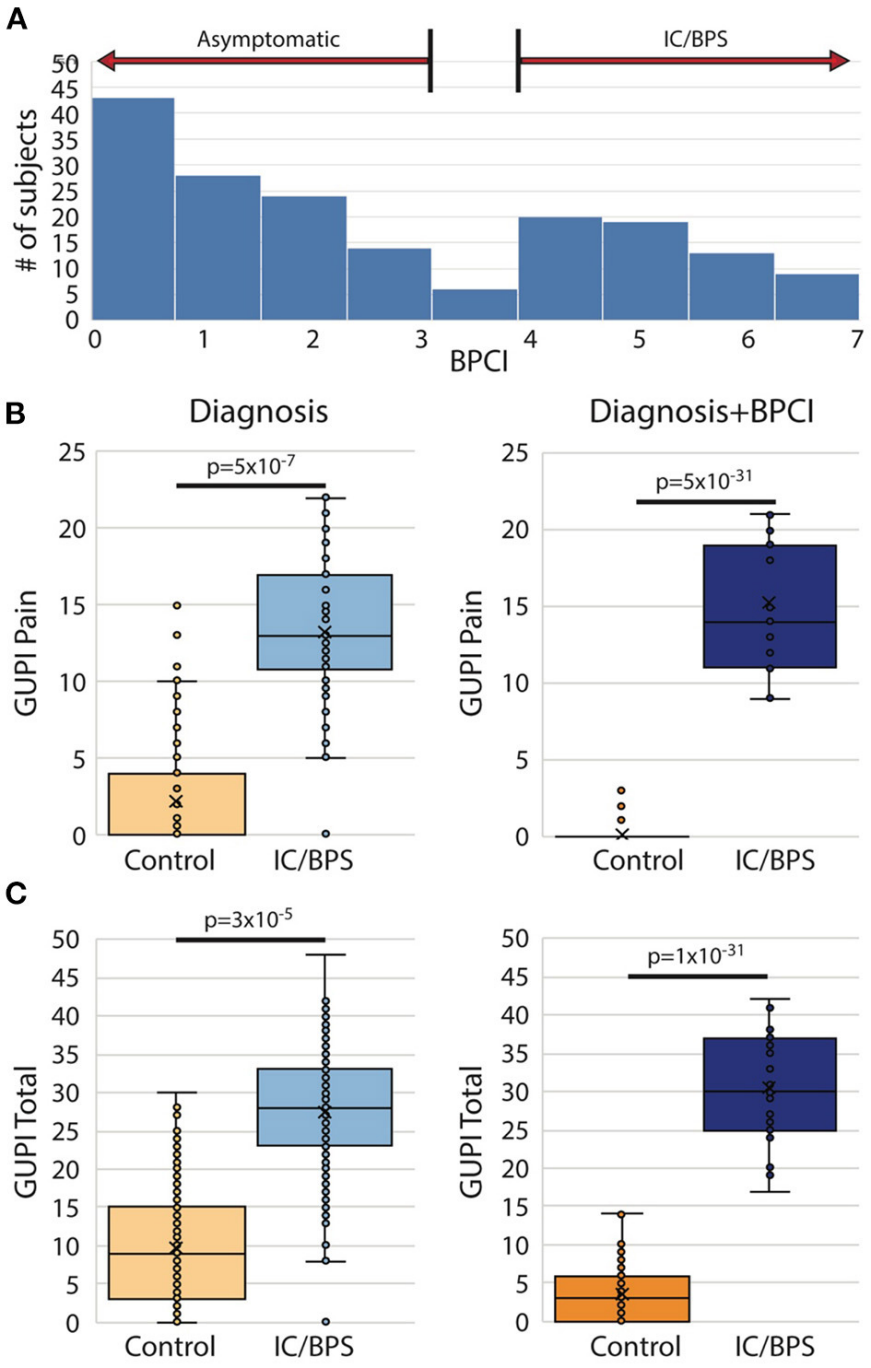
Bladder pain composite index (BPCI) defines more homogeneous IC/BPS and control populations. **(A)** BPCI score distribution for 521 subjects with and without urinary symptoms recruited as possible participants revealed a clear division between patients with and without bladder pain and was used to define our study populations for this proposal. **(B,C)** Use of clinical diagnosis of IC/BPS results in substantial symptomatic overlap in **(B)** pain severity (fGUPI Pain domain) and **(C)** overall urinary symptoms (fGUPI total score) with subjects identified as controls (left), while separation based on a BPCI>4 provides more homogeneous, distinct populations (right).

From the original group of 521 potential subjects with genitourinary pain, 183 (35%) were assigned a diagnosis of IC/BPS. Although pain levels in this group were significantly elevated in comparison to control subjects, identification of subject groups by diagnosis was not sufficient to separate IC/BPS patients from controls symptomatically (Figure 1A). In addition, approximately 25% of patients included in the control group expressed some bother associated with urinary/pelvic symptoms, as determined by an fGUPI quality of life subscale >4. As such contamination between cases and controls could confound the description of unique pain phenotypes, we selected only the 145 pre-menopausal women of the 183 who bore a clinical diagnosis of IC/BPS or bladder pain *and* also scored 4 or higher on the BPCI, a measure of pain selectively associated with the bladder. Sixty-nine age-matched, asymptomatic subjects with BPCI < 3 were selected from the potential subject pool as controls. As the hormonal changes in the peri- and postmenopausal period add an additional level of complexity and obscure the relation of symptomatology to bladder pain, only pre-menopausal women were included. This selection avoids the complicating comorbidities and co-existing genitourinary symptoms, such as detrusor underactivity and overactive bladder, common in post-menopausal women. The combination of narrow inclusion criteria using clinician diagnosis in conjunction with objective scoring on the BPCI corrected for confounders within and between study groups to reliably identify IC/BPS subjects *and* separate symptomatic patients from controls (Figures 1B,C). The baseline characteristics of the included participants are summarized in Table 1.

**Table 1:** Patient demographics.

	**IC/BPS** **(*n* = 145)**	**Controls** **(*n* = 69)**	***P*-values**
Age: years (SD)	31.8 (6.9)	34.1 (6.4)	0.08
Average prescription meds: number (SD)	0.8 (1.1)	0.5 (0.8)	0.28
BMI: kg/m^2^ (SD)	25.4 (7.1)	26.3 (5.5)	0.63
Hormonal birth control: percent (n)	23.5% (34)	23.2% (16)	0.96
**Comorbidities: percent (n)**			
Anxiety	13.1% (19)	5.7% (4)	0.06
Depression	6.9% (10)	5.7% (4)	0.75
Endometriosis	2.1% (3)	0% (0)	0.08
Fibromyalgia	2.1% (3)	0% (0)	0.08
GERD	0.7% (1)	4.3% (3)	0.16
Hyperlipidemia	0.7% (1)	4.3% (3)	0.16
IBS	10.3% (15)	2.8% (2)	*0.02
Migraine	4.1% (6)	2.8% (2)	0.63
Nephrolithiasis	1.4% (2)	7.2% (5)	0.07
PCOS	2.1% (3)	1.4% (1)	0.73

GERD, gastroesophageal reflux disease; IBS, irritable bowel syndrome; PCOS, polycystic ovary syndrome*.

### Unsupervised Clustering of IC/BPS Subjects Into Unique Clusters

**Figure 2:**
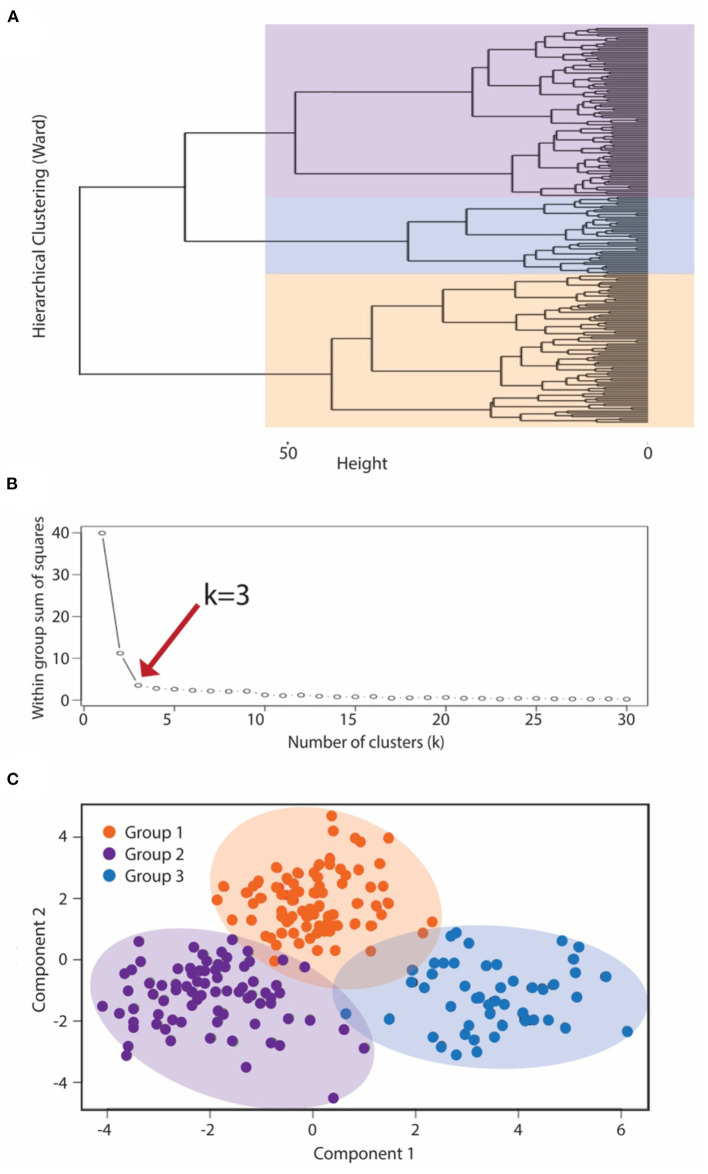
K means clustering to subcategorize pre-menopausal female subjects with IC/BPS. To classify IC/BPS patients into phenotypes, we utilized a k-means algorithm of unsupervised clustering for an independent group of subjects. **(A)** Dendrogram visualizing the order and distances of subjects for merges during hierarchical clustering by Ward's method, with the overlaid colors representing the three-group solution as the optimal *k*. **(B)** The number of phenotypes (clusters), k, was also determined by the minimum number of groups yielding the most meaningful cluster profiles according to the within group sum of squares method (“elbow” method). **(C)** Centroid plot for the three-cluster solution by *K*-means clustering of symptomatic profiles demonstrated clear separation of these clusters into three groups, corresponding to the BPS group (purple), MFP group (orange), and NUPP group (blue).

While IC/BPS is defined as *bladder pain* in the absence of infection or other organic pathology, difficulties discriminating pelvic visceral sensation and a lack of well-defined language describing pelvic pain complicate IC/BPS diagnosis and subclassification. As a quantitative approach to identifying more homogeneous IC/BPS subpopulations, we used *K*-means clustering, an unsupervised machine learning approach, to recognize distinct phenotypes based on the patient-reported measures examining GU symptomatology from the 145 IC/BPS subjects. *K*-means clustering necessitates defining *k*, the fixed number of clusters you anticipate within the dataset. To define this value, we first applied hierarchical Ward's clustering to the symptomatic assessment instruments alone, revealing that a three-cluster solution best fit the dataset of patients with perceived bladder pain (Figure 2A). To validate this choice of cluster groups, we employed the elbow method to identify the number of clusters after which adding more groups provides little improvement in the model. Using this approach (Figure 2B), the clinical data also confirmed that clustering into three groups yielded the most meaningful number of cluster profiles. We then used *K*-means clustering analysis (17) to divide subjects into three (*k*) clinical phenotypes based only on the patient responses to symptomatic questionnaires. This cluster solution assigned 56 subjects to group 1, 31 patients to group 2, and 58 patients to group 3. The meaningful separation of these groups by symptomatic assessment measures was visually confirmed by principal coordinate analysis using Bray-Curtis dissimilarity measures (Figure 2C).

### Baseline Clusters Show Distinct Bladder Pain Phenotypes

**Figure 3:**
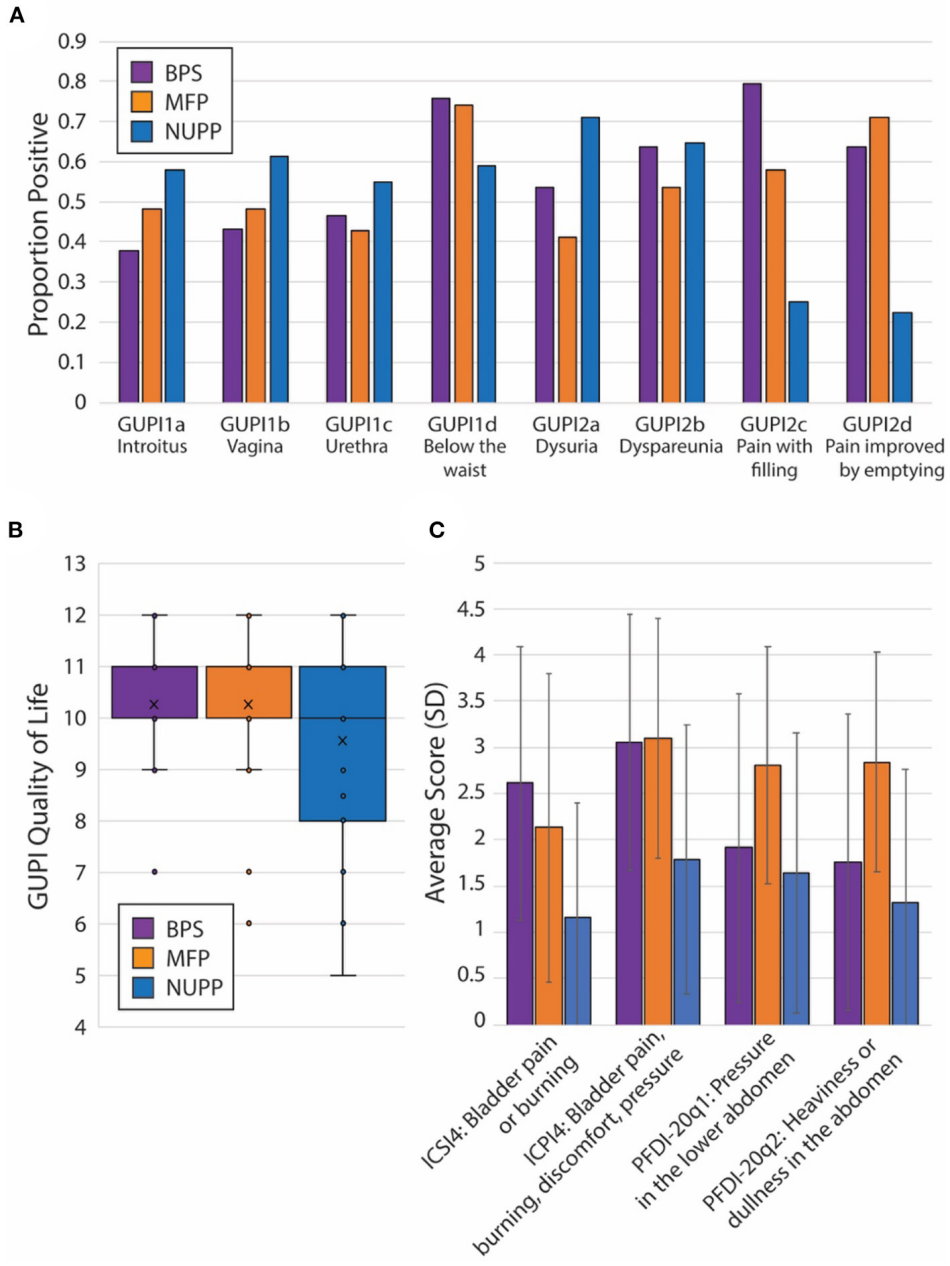
Unique features of each IC/BPS phentoypic cluster. **(A)** Individual proportions of IC/BPS subjects endorsing pain symptoms by location (fGUPI1) and activity (fGUPI2) by phenotype. **(B)** Box and whisker plot displaying the composite quality of life score on the fGUPI for each phenotype in the inset legend. **(C)** Mean scores for four independent measures of bladder-related pain, pressure or discomfort are shown by phenotypic group. Error bars display the standard deviations. Significances for all pairwise comparisons are listed in Table 2.

To explore the face validity of the unsupervised clustering, we combined a review of the symptomatic questionnaire scores (Table 2) with a qualitative chart review of the phenotyped subjects. Regardless of phenotype, almost all patients categorized as IC/BPS displayed elevated pain levels on a visual analog scale (fGUPI4) as well as significant urinary frequency (ICSI2, ICPI1) and discomfort below the waist (fGUPI1d). The first group described a pattern of bladder-specific pain (ICSI4) that was aggravated by bladder filling (fGUPI2c) and relieved by emptying (fGUPI2d) (Figure 3A), a constellation of symptoms we dubbed bladder-specific pain symptoms (BPS). Of all the groups, this group most commonly expressed sensitivities of their bladder pain symptoms to dietary triggers, such as acidic foods or caffeine, on chart review, although this domain was not assessed in all subjects.

**Table 2:** Geometric means for patient scores on individual symptomatic questions.

**Question**	**Symptom feature**	**MFP** **(Std Dev)**	**BPS** **(Std Dev)**	**NUPP** **(Std. Dev)**	***P*** **-values**		
					**MFP vs. BPS**	**MFP vs. NUPP**	**BPS vs. NUPP**
ICSI1	Strong need to void with no warning	2.98 (±1.41)	3.16 (±1.48)	1.04 (±1.14)	0.59	<0.001	<0.001
ICSI2	Urinary frequency within 2 h	4.19 (±1.04)	4.29 (±0.96)	2.16 (±1.37)	0.65	<0.001	<0.001
ICSI3	Nighttime urination	2.95 (±1.36)	1.97 (±1.15)	1.10 (±1.11)	<0.001	<0.001	<0.001
ICSI4	Pain or burning in the bladder	2.13 (±1.67)	2.61 (±1.48)	1.16 (±1.23)	0.16	<0.001	<0.001
ICSI Total	**ICSI 1–4**	12.45 (±3.76)	11.95 (±2.90)	5.45 (±3.04)	0.49	<0.001	<0.001
ICPI1	Frequent daytime urination	3.12 (±1.02)	3.33 (±0.62)	1.46 (±1.14)	0.26	<0.001	<0.001
ICPI2	Getting up at night to urinate	3.10 (±1.08)	2.53 (±1.19)	0.92 (±1.14)	0.03	<0.001	<0.001
ICPI3	Need to urinate with little warning	2.84 (±0.90)	2.40 (±1.20)	0.88 (±1.11)	0.08	<0.001	<0.001
ICPI4	Bladder burning, pain, discomfort, or pressure	3.10 (±1.30)	3.05 (±1.38)	1.75 (±1.46)	0.88	<0.001	<0.001
ICPI Total	**ICPI 1–4**	12.16 (±2.55)	11.31 (±2.47)	5.05 (±3.02)	0.12	<0.001	<0.001
OLS	**ICSI + ICPI**	24.61 (±5.62)	23.26 (±4.92)	10.51 (±5.67)	0.24	<0.001	<0.001
OABq2	Uncomfortable urge to urinate	4.77 (±1.38)	4.76 (±1.11)	2.33 (±1.22)	0.95	<0.001	<0.001
OABq3	Sudden urge to urinate with no warning	4.19 (±1.70)	3.74 (±1.62)	1.92 (±1.17)	0.22	<0.001	<0.001
OABq4	Accidental loss of small amounts of urine	3.97 (±1.87)	2.05 (±1.42)	1.89 (±1.26)	<0.001	<0.001	<0.001
OABq5	Nighttime urination	4.45 (±1.69)	3.67 (±1.66)	2.02 (±1.05)	0.04	<0.001	<0.001
OABq6	Waking at night to urinate	4.55 (±1.46)	3.93 (±1.53)	2.20 (±1.17)	0.07	<0.001	<0.001
OABq8	Urine loss associated with strong urgency	3.87 (±1.89)	2.59 (±1.85)	1.41 (±0.97)	0.002	<0.001	<0.001
OABq SF	**OABq 2–6, 8**	25.81 (±7.34)	20.74 (±5.80)	11.79 (±4.18)	<0.001	<0.001	<0.001
fGUPI1A	Discomfort at the entrance to the vagina	0.48	0.38	0.58	0.27	0.38	0.07
fGUPI1B	Discomfort in the vagina	0.48	0.43	0.61	0.59	0.25	0.10
fGUPI1C	Discomfort in the urethra	0.43	0.47	0.55	0.69	0.29	0.46
fGUPI1D	Discomfort below the waist	0.74	0.76	0.59	0.86	0.16	0.05
fGUPI2A	Pain or burning during urination	0.41	0.53	0.71	0.19	0.11	0.18
fGUPI2B	Pain or discomfort with sexual intercourse	0.54	0.63	0.64	0.44	0.39	0.97
fGUPI2C	Pain or discomfort as your bladder fills	0.58	0.79	0.25	0.03	0.002	<0.001
fGUPI2D	Pain or discomfort relieved by voiding	0.71	0.63	0.22	0.50	<0.001	<0.001
fGUPI3	How often was your pain	3.90 (±1.10)	3.67 (±1.13)	2.98 (±1.15)	0.36	<0.001	0.002
fGUPI4	Average pain or discomfort	6.39 (±1.76)	6.13 (±1.52)	2.98 (±1.61)	0.49	0.01	0.02
fGUPI Pain	**Total fGUPI 1–4**	14.66 (±4.24)	14.45 (±3.67)	10.11 (±3.42)	0.26	<0.001	<0.001
fGUPI5	Sensation of not emptying your bladder	3.37 (±1.37)	2.65 (±1.67)	1.67 (±1.64)	0.04	<0.00	0.002
fGUPI6	Urinate again within 2 h	3.60 (±1.16)	4.05 (±1.02)	2.01 (±1.42)	0.06	<0.001	<0.001
fGUPI Urinary	**Total fGUPI 5–6**	6.96 (±2.30)	6.71 (±2.08)	3.70 (±2.62)	0.59	<0.001	<0.001
fGUPI7	Impact on activities	2.29 (±0.64)	1.89 (±1.04)	1.27 (±1.14)	0.06	<0.001	0.002
fGUPI8	Distraction by symptoms	2.65 (±0.75)	2.66 (±0.61)	2.18 (±0.92)	0.94	0.02	0.001
fGUPI9	Satisfaction with current symptoms	5.32 (±0.83)	5.01 (±0.97)	4.33 (±1.62)	0.13	0.002	0.007
fGUPI Bother	**Total fGUPI 7–9**	10.26 (±1.77)	9.56 (±1.90)	7.78 (±3.04)	0.09	<0.001	<0.001
fGUPI Total	**Total fGUPI 1–9**	31.87 (±6.67)	30.72 (±5.22)	21.59 (±6.21)	0.13	<0.001	<0.001
PFDI20-1	Pressure in the lower abdomen	2.81 (±1.78)	1.91 (±1.67)	1.64 (±1.52)	0.01	<0.001	0.36
PFDI20-2	Heaviness or dullness in the abdomen	2.83 (±1.60)	1.76 (±1.60)	1.32 (±1.44)	0.001	<0.001	0.13
PFDI20-3	Vaginal bulge	0.76 (±1.40)	0.34 (±0.87)	0.54 (±1.16)	0.06	0.39	0.32
PFDI20-4	Splint to defecate	1.58 (±1.13)	0.40 (±0.92)	0.57 (±0.99)	<0.001	<0.001	0.33
PFDI20-5	Feeling of incomplete emptying	3.39 (±1.22)	1.62 (±1.57)	1.07 (±1.25)	<0.001	<0.001	0.04
PFDI20-6	Splinting to void	0.87 (±0.75)	0.12 (±0.59)	0.02 (±0.13)	0.001	<0.001	0.21
POPDI-6	**Total PFDI 1–6**	51.0 (±18.7)	25.7 (±15.0)	21.5 (±18.9)	<0.001	<0.001	0.19
PFDI20-7	Straining to have a bowel movement	2.26 (±1.18)	0.76 (±1.13)	1.07 (±1.21)	<0.001	<0.001	0.16
PFDI20-8	Tenesmus	2.32 (±1.56)	0.74 (±1.09)	1.18 (±1.31)	<0.001	<0.001	0.05
PFDI20-9	Loss of formed stool	1.03 (1.35)	0.02 (±0.13)	0.02 (±0.13)	<0.001	<0.001	0.98
PFDI20-10	Loss of liquid stool	1.56 (±1.62)	0.23 (±0.66)	0.16 (±0.56)	<0.001	<0.001	0.50
PFDI20-11	Flatal incontinence	2.03 (±1.43)	0.21 (±0.69)	0.39 (±0.98)	<0.001	<0.001	0.25
PFDI20-12	Pain with bowel movements	1.29 (±1.51)	0.14 (±0.43)	0.43 (±0.81)	<0.001	<0.001	0.02
PFDI20-13	Urgency to have a bowel movement	2.75 (±1.03)	0.70 (±1.08)	0.59 (±1.01)	<0.001	<0.001	0.57
PFDI20-14	Rectal prolapse	1.37 (±1.52)	0.05 (±0.30)	0.07 (±1.03)	<0.001	<0.001	0.79
CRADI-8	**Total PFDI 7–14**	45.7 (±16.6)	8.9 (±4.8)	12.2 (±12.0)	<0.001	<0.001	0.03
PFDI20-15	Frequent urination	3.05 (±1.39)	2.83 (±1.22)	1.32 (±1.33)	0.44	<0.001	<0.001
PFDI20-16	Urine leakage with urgency	2.6 (±1.63)	1.24 (±1.48)	0.41 (±0.85)	<0.001	<0.001	<0.001
PFDI20-17	Urine leakage related to cough, laugh, sneeze	1.98 (±1.47)	1.05 (±1.33)	1.00 (±1.21)	0.003	0.001	0.82
PFDI20-18	Small amounts of urine loss	2.46 (±1.47)	0.86 (±1.22)	0.55 (±1.06)	<0.001	<0.001	0.15
PFDI20-19	Difficulty emptying your bladder	2.02 (±1.65)	1.28 (±1.46)	0.73 (±1.17)	0.03	<0.001	0.03
PFDI20-20	Pain or discomfort in lower abdomen	3.05 (±1.04)	2.18 (±1.59)	1.66 (±1.61)	0.007	<0.001	0.08
UDI-6	**Total PFDI 15–20**	63.2 (±4.72)	39.3 (±17.6)	23.6 (±16.7)	<0.001	<0.001	<0.001
Age	Years	31.34 (±19.7)	31.53 (±7.18)	37.8 (±6.3)	0.13	<0.001	0.001

*fGUPI 1A-2D are yes/no questions; the responses are shown as proportions. All other questions are Likert scales, for which the means and standard deviations are shown. ICSI, Interstitial Cystitis Symptom Index; ICPI, Interstitial Cystitis Problem Index; OLS, O'Leary-Sant Indices total score; OABqSF, Overactive Bladder Questionnaire Short Form; fGUPI, Genitourinary Pain Index; PFDI-20, Pelvic Floor Distress Inventory Short Form; POPDI-6, Pelvic Organ Prolapse Distress Inventory 6; CRADI-8, Colorectal-Anal Distress Inventory 8; UDI-6, Urinary Distress Inventory Short Form. Significant p-values are indicated in red*.

The second group exhibited persistent, non-cyclic pelvic pain unrelated to bladder filling or emptying, which we designated non-urologic pelvic pain (NUPP). In these patients, the fGUPI revealed higher levels of pain localized to the vagina and urethra (fGUPI1a,b,c), coexisting with both dyspareunia and dysuria (fGUPI2a,b) (Figure 3A). NUPP subjects typically had lower scores on questions regarding bladder pain and discomfort (ICSI4 and ICPI4) and denied pain related to the voiding cycle (fGUPI2c,d). On chart review, the NUPP group tended to describe a more diffuse pelvic discomfort, with more specific pain localized to the urethra and distal vagina/introitus. All of the groups exhibited a significant impact on quality of life and dissatisfaction with their current symptoms, but the NUPP group appeared the least impacted of the three groups (fGUPI QOL subscale 7.78 ± 3.04 for NUPP vs. 9.56 ± 1.90 BPS and 10.26 ± 1.77 MFP) (Figure 3B).

The third group, designated as myofascial pain (MFP), exhibited a wide range of symptoms that were elevated, such as urinary frequency, bladder discomfort, and pelvic pressure, in comparison to controls. This group was distinguished by significantly more defecatory symptoms (PFDI20 questions 7–14), an increased sensation of incomplete emptying of the bladder (PFDI-20q5, fGUPI5), and small amounts of urine leakage (PFDI-20q18), typically without awareness. While these subjects expressed similarly elevated “pain below the waist” (fGUPI1d) as the other groups, they had the lowest proportions of pain in other pelvic locations specified on questions 1 and 2 on the fGUPI, which specify types of pain in the pelvis through a set of eight yes/no questions (Figure 3A). However, on the PFDI-20, a questionnaire designed to assess for symptoms of pelvic organ prolapse (POP), the MFP group consistently demonstrated higher symptomatic bother than the other groups, except in their endorsement of a vaginal bulge (PFDI-20q3). This group had higher scores than those historically described for patients with POP (16), but without the discriminatory feature of a vaginal bulge or evidence of prolapse on physical exam (16). Instead, chart review showed obvious findings of myofascial pain on physical exam, manifest as either tenderness to palpation of or distinct trigger points in the levator muscles and hip flexors (primarily obturator internus) (22).

Subjects in this group had nearly identical pain levels, urinary symptoms and severity, and quality of life impact as the BPS group. The major difference in their symptoms was the associated defecatory symptoms. The symptomatic questionnaires, however, include multiple questions describing the nature of the pain (Figure 3C). ICPI4 asks about “burning, pain, discomfort or pressure” in the bladder, while ICSI4 specifies “pain or burning” only. MFP subjects tended to score higher on ICSI4 than on ICPI4. The MFP group has significantly lower proportions of subjects who endorsed pain with bladder filling (fGUPI2c) than the BPS group but endorsed similar pain to the BPS group “below the waist” (fGUPI1d). Questions 1 and 2 on the PFDI-20, respectively, describe “pressure” and “heaviness or dullness”; the MFP subjects scored significantly higher on these two questions than either the BPS or NUPP groups. This pattern of scores suggests that the MFP group experiences pelvic discomfort more accurately described as a pressure or discomfort, while the BPS group describes more frank pain, particularly related to bladder filling.

### Cluster Stability

To ensure the stability of our cluster assignment, we resampled (500) with replacement (bootstrapping) a large number of replications (10,000) and identified the cluster assignments for each iteration. From these we computed the percent observed agreement (Rand Index) and percent overlap (Jaccard Coefficient), for which values over 0.7 indicate good cluster stability. The Rand indices were 0.86 (95%CI: 0.85–0.87) for BPS, 0.85 (95%CI: 0.084–0.86) for NUPP and 0.75 (95%CI 0.74–0.76) for MFP, demonstrating high percent observed agreement of the bootstrapped samples. The clusters had Jaccard coefficients of 0.68 (95%CI: 0.67–0.69) for BPS, 0.55 (95%CI: 0.53–0.57) for NUPP and 0.49 (95% CI: 0.47–0.52) for MFP. These measures of agreement suggest that for the NUPP and MFP clusters, the high observed percent agreement was driven by the absence of observations being assigned to these clusters. However, the BPS cluster showed stability based on both percent agreement and percent overlap. Overall, the cluster stability measures indicate that only the BPS cluster would be likely to be detected using a similar definition in a different sample.

### Measures Examining Each Symptom Complex Can Discriminate Bladder Pain Phenotypes

**Figure 4:**
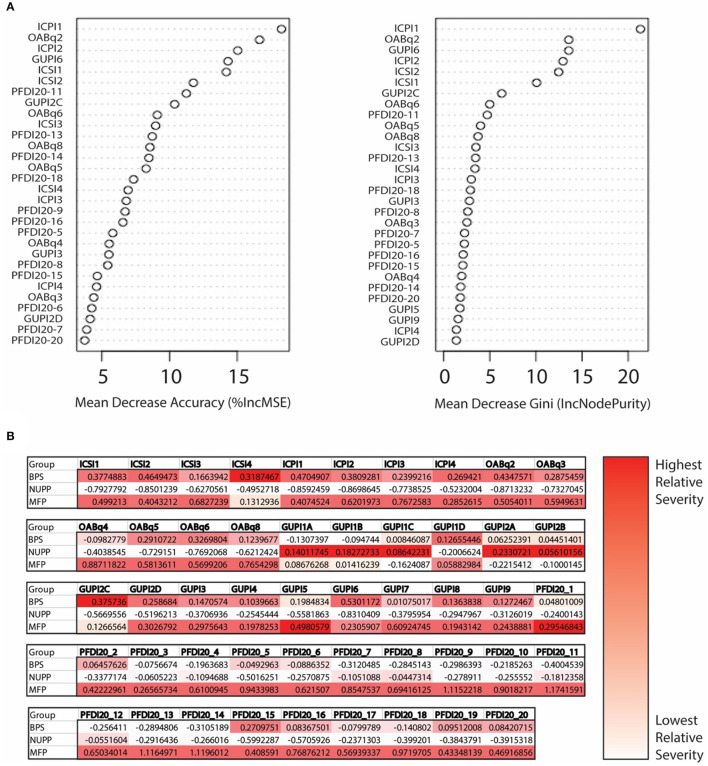
Determination of important clinical features defining the unique IC/BPS phenotypes. **(A)** Variable importance plot demonstrating how important each variable is in classifying the data. Mean decrease Accuracy expresses how much accuracy the model loses by excluding individual variables. The mean decrease in Gini coefficient measures how much each variable contributes to generating homogeneous nodes, with higher values indicating greater importance in the model. **(B)** Mean scores for each phenotype are expressed as Z scores to provide a normalized distribution of scores relative to the mean of the overall population regardless of individual item scale. These were then expressed in heat maps for each question to provide a visual representation of scores uniquely associated with a single phenotype, with the highest scoring questions shown in the darkest red.

**Figure 5:**
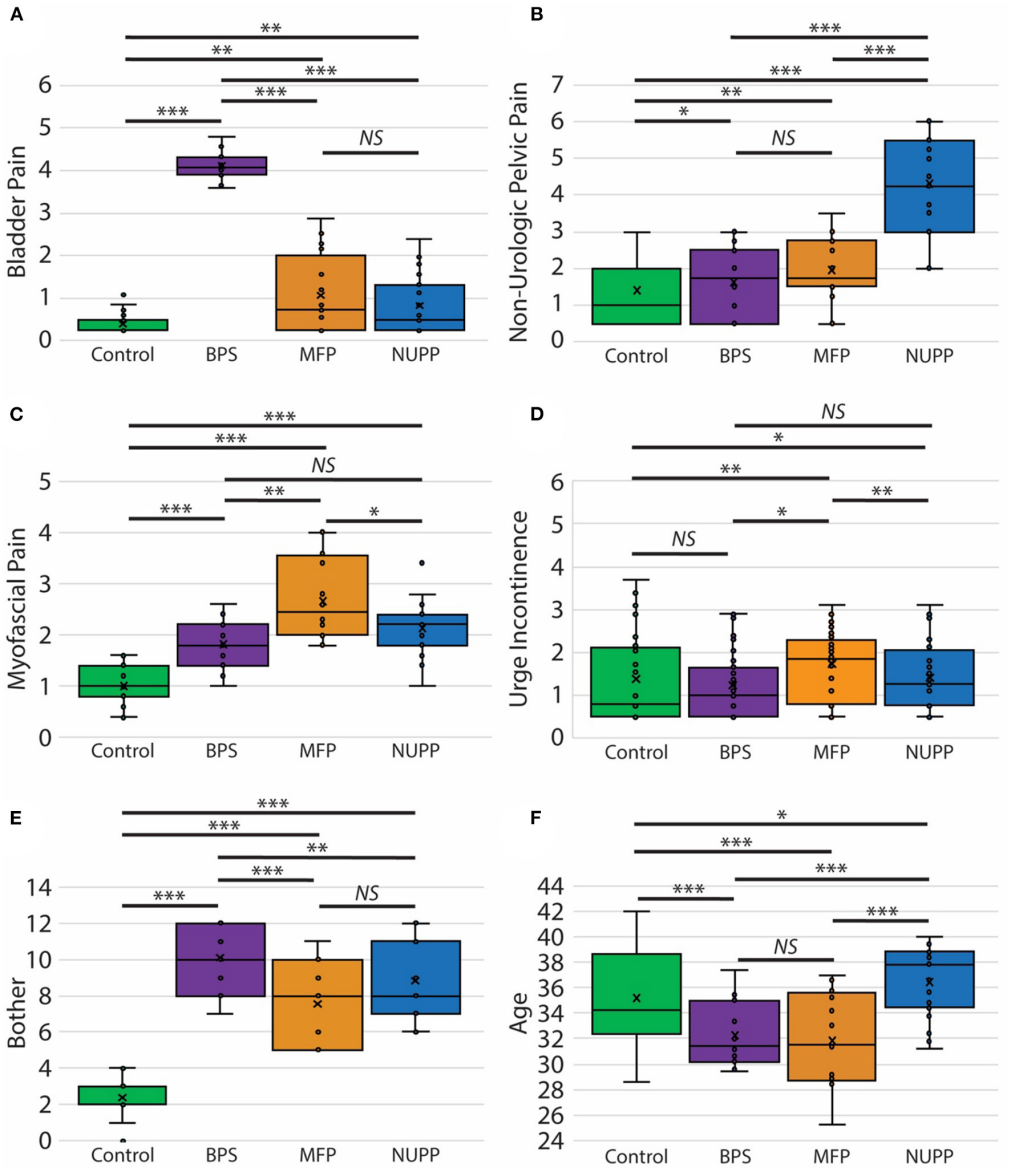
Clinical features of pelvic pain patients identified by unsupervised clustering. Measures of the phenotypic characteristics of the presumptive IC/BPS clusters are plotted as box and whisker plots. Subjects exhibited distinct associated pain symptoms. The BPS group (purple) was homogenously high for bladder pain related to bladder filling **(A)**, while the NUPP group (blue) demonstrated pelvic pain focused on the urethra and vagina unrelated to urination **(B)**. The MFP group (orange) had features of myofascial pain **(C)**. None of these groups exhibited significant urgency incontinence **(D)** and were all significantly bothered by their symptoms in comparison to controls **(E)**. The BPS and MFP groups were significantly younger than controls or NUPP patients **(F)**. ^***^p < 0.0001, ^**^p < 0.005, and ^*^p < 0.05.

We next utilized a random forest model (23) to identify important features used to classify subjects in the *K*-means algorithm (Figure 4A). From the 20 questions with the largest impact on phenotypic classification, we then selected the questions with largest association with specific phenotypic groups, as expressed in a heat map of the scaled values (Figure 4B). These discriminatory questions were combined to express the relative severity of these symptom domains for each phenotypic group, plotted in box and whisker plots for the three IC/BPS phenotypes in comparison to controls (Figure 5). While all IC/BPS subjects exhibited highly elevated symptomatic bother (Figure 5E), the severity of each symptom complex differed greatly. For the BPS group, a bladder pain measure was generated from the weighted composite of ICSI4 (bladder pain or burning), fGUPI2c (pain with bladder filling), and fGUPI6 (urinary frequency). These specific features were highly restricted to the BPS group (Figure 5A). For the NUPP group, a measure of non-urologic pelvic pain was generated from the sum of fGUPI1a, b, and c (pain in the introitus, vagina, and urethra, respectively) and fGUPI2a and b (dysuria and dyspareunia, respectively). Using this measure, the NUPP group was easily distinguished from the other pain groups and controls (Figure 5B). To represent the symptoms of the MFP group, we incorporated the scaled scores for fGUPI5 and PFDI-20q5 (sensation of incomplete emptying), PFDI-20q1 (abdominal pressure), and PFDI-20q7 (straining to defecate) into a single measure. While this constellation of symptoms, bladder pressure and incomplete elimination symptoms, was most elevated in the MFP group, varying degrees of these symptoms were present in all three groups over the baseline seen in asymptomatic controls (Figure 5C). The subjects only infrequently complained of symptoms not classically associated with IC/BPS, such as urgency incontinence measured by the urge incontinence composite index (UICI) (7). While incontinence symptoms were highest in the MFP group, the scores for all groups remained low overall (Figure 5D). Interestingly, the BPS and MFP groups were significantly younger than NUPP subjects and asymptomatic controls (Figure 5F), suggesting an association of these phenotypes with younger age.

### Overlap Between Phenotypes Within the IC/BPS Population

**Figure 6:**
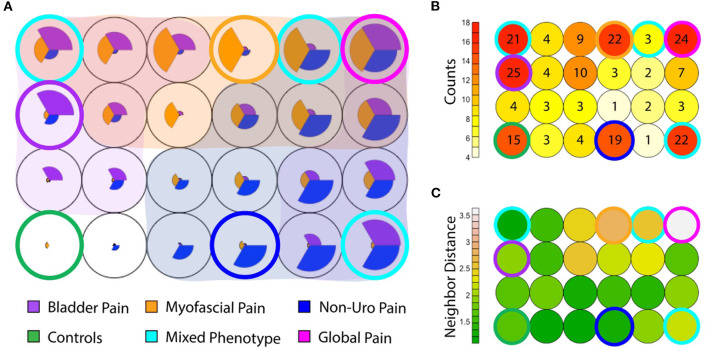
Coexisting symptom domains determined by Kohonen self-organizing map (SOM). **(A)** The SOM groups subjects (including IC/BPS subjects and asymptomatic controls) into 24 (6 × 4) bins. Within each, the size of each pie slice expresses the average measure score for that subgroup after scaling to normalize values. Asymptomatic controls are at the inferior left corner (green ring), in which no pie slices are seen. The canonical phenotypes, expressing only a single symptom cluster are ringed in blue for NUPP, orange for MFP, and purple for BPS. Other combinations of phenotypes are shaded by an overlay of the respective group color when that symptom profile was expressed within that group. The most common mixed phenotypes are ringed in cyan. The global pain phenotype, in which all three symptom types were highly expressed, is ringed in magenta. **(B)** The numbers of patients within each of the groups in A are expressed in a heat map, with the relative position of each bin corresponding to the layout seen in **(A)**. The number indicates the absolute number of subjects in each bin. Most subjects reside in the control and the ringed phenotypic groups, with a substantial minority present in subgroups expressing more than one phenotype. **(C)** The similarity of each of the symptom patterns to the neighboring bin in Euclidean distance is expressed as a heat map (each bin shading from green to white). For example, the MFP phenotype ringed in orange is highly dissimilar to the surrounding symptom combinations, but the global pain phenotype ringed in magenta is the most dissimilar from all other groups, demonstrating the largest Euclidean distance to its neighbors.

While most IC/BPS subjects displayed only one predominant symptom cluster, some individuals exhibited features of more than one group. These overlapping symptoms raised the question of whether these phenotypes could manifest independently. We utilized a Kohonen SOM (24), a type of neural network trained using unsupervised learning, to explore the relative expression of the clinical features defining each phenotype across the cohort, using the symptomatic measures defined above. Graphically, patients are clustered into an arbitrary number of “bins,” each represented in the figure by a circle, and the relative expression of each symptom domain (bladder pain, myofascial pain and non-urologic pelvic pain as defined in the measures above) for that bin is represented by the size of each pie slice within the circle (Figure 6A). Given the low number of subjects, the number of bins was kept small (*n* = 24 in a 6 × 4 grid) to allow for meaningful groups of similar patients to emerge. Only a portion of the total subjects (*n* = 81/214; 38%) including the pain groups and controls fell into a “pure” phenotype category (asymptomatic controls, BPS, NUPP, and MFP). These “pure” phenotypes, in which a single symptomatic feature (or “pie slice”) was clearly dominant, are designated by a colored ring surrounding the bin. The red heat map in Figure 6B represents the number of patients in each bin corresponding in space to those depicted in Figure 6A. The remaining 79 IC/BPS patients fell into bins expressing combinations of two or more of these symptom clusters (cyan), including a ‘global pain' phenotype (magenta) in which subjects demonstrated high levels of all three types of pain symptoms. Interestingly, almost half of the control subjects expressed symptoms placing them in bins with other symptomatic patients, although they did not complain of significant bother associated with these symptoms. The similarity of each of the symptom patterns (each bin shading from green to white) to the neighboring bin's pattern is also expressed as a heat map (in green) in Figure 6C. The global pain phenotype (upper right in grid) exhibited the largest symptomatic difference from the other groups. The pure MFP (ringed in orange) and MFP-dominant phenotypes were more dissimilar to the surrounding symptom combinations than the BPS and NUPP phenotypes were to each other, mirroring the hierarchical clustering in Figure 2.

### Unique IC/BPS Phenotypes Demonstrate Different Responses to IC/BPS Therapies

**Figure 7:**
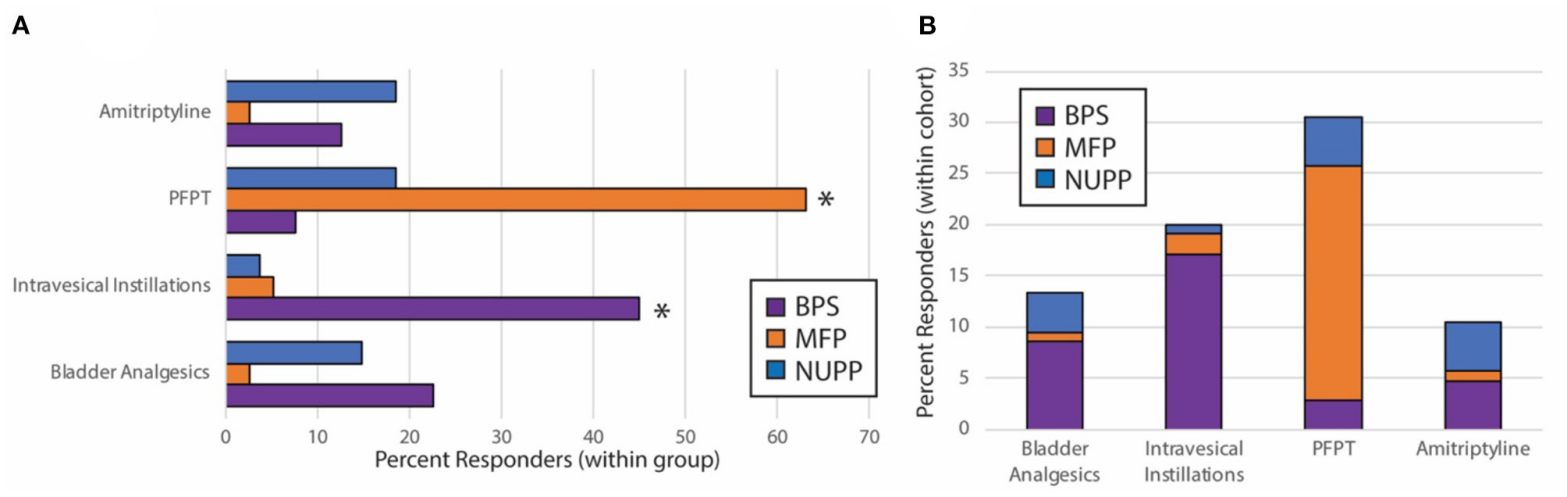
Subjective treatment responses vary by phenotype. Chart review of subjects in the BPS, MFP, and NUPP groups was used to assess binary responsivity (responded yes/no) to several typical therapies prescribed for IC/BPS patients. **(A)** Responder rates for patients were expressed as a percentage of each individual phenotype. The starred treatment groups were statistically different from both of the other groups (^*^p < 0.001 for pairwise comparisons with both other phenotypes). **(B)** The proportion of responders within the total population was expressed for each therapy, demonstrating that the majority of responders to the bladder-directed therapies (bladder analgesics and intravesical instillations) were BPS patients, while the majority of responders to PFPT were MFP subjects. Responses were only assessed for those subjects who attempted the designated therapies.

After observing segregation of the original IC/BPS population into phenotypes with divergent clinical features, we hypothesized each would respond differently to IC/BPS therapies. We performed retrospective chart review of subjects for each group to determine patient-perceived responder rates for four common IC/BPS therapies: oral bladder analgesics, intravesical instillations, pelvic floor physical therapy (PFPT), and oral amitriptyline (Table 3, Figure 7). Of patients who had attempted each therapy, the BPS group responded best to bladder-directed therapies, such as bladder analgesics and intravesical instillations. The response to intravesical instillations was significantly better in the BPS phenotype than in either the NUPP or MFP groups (*p* < 0.001 for both). We observed that the NUPP group consistently demonstrated low response rates to all therapies. In contrast, MFP subjects responded well to PFPT, with almost 80% of those who could be assessed responding positively to this therapy (79 vs. 12% for NUPP and 9% for BPS; *p* < 0.0001 for both). Unfortunately, the number of patients in this group that were not seen in follow-up after PFPT was enriched due to clinic closures associated with the pandemic. Regardless, these findings are suggestive that IC/BPS phenotypes can be identified that require different therapeutic strategies to achieve symptomatic control.

**Table 3:** Responders to typical IC/BPS therapies by phenotypic cluster.

**Phenotype**	**Oral bladder** **analgesics^**†**^**	**Intravesical** **instillations^**‡**^**	**Pelvic floor** **physiotherapy**	**Amitriptyline**	**Total patients** with follow-up^*^
BPS	9 (*n* = 12)	18 (*n* = 20)	3 (*n* = 4)	5 (*n* = 8)	40 (44 therapies attempted)
MFP	1 (*n* = 6)	2 (*n* = 4)	23 (*n* = 27)	1 (*n* = 2)	38 (39 therapies attempted)
NUPP	4 (*n* = 13)	1 (*n* = 10)	5 (*n* = 21)	5 (*n* = 15)	27 (59 therapies attempted)
Responders	14 (*n* = 31)	21 (*n* = 34)	31 (*n* = 52)	11 (*n* = 25)	105

*Patients with a positive response are indicated for each therapeutic approach by phenotype. The total numbers of patients attempting each therapy are denoted in parentheses*.

† *Oral analgesics included phenazopyridine, Urogesic-Blue™ (hyoscyamine, methenamine, methylene blue, and sodium biphosphate), and Uribel™ (hyoscyamine, methenamine, methylene blue, phenyl salicylate, sodium phosphate) or other formulations with similar composition*.

‡ *Standard bladder instillation included lidocaine, sodium bicarbonate, heparin, and triamcinolone*.

* *The number of attempted therapies for each group are greater than the total number of patients with follow up as multiple patients per group attempted more than one therapy*.

## Discussion

Deeper clinical characterization of female premenopausal patients with the subjective sensation of bladder pain reveals three distinct phenotypes which suggest divergent sources of perceived pain. Each of the phenotypes had recognizably different clinical features as well as different responses to the various treatments frequently employed for IC/BPS patients, data which support differing pathophysiologies underlying these phenotypes.

For many chronic pain conditions, such as IC/BPS, the condition is defined by a process of exclusion of other organic pathologies. The exclusion of other conditions does not, however, mean that the remaining affected individuals are homogeneous. Depending on the inclusion criteria, patients endorsing the sensation of bladder pain or discomfort may include multiple symptomatologies: true pain deriving from aberrant sensation in the bladder, other forms of pelvic pain referred to the bladder, centralized or systemic allodynia including features of pain ascribed to the bladder, or other localized pelvic pain misattributed to, but unrelated to the bladder. These different symptom origins may be described similarly by patients but are unlikely to represent a single clinical condition or respond to identical treatment approaches. While there is growing consensus that the recognition of Hunner's lesions in IC/BPS should prompt different considerations for medical and surgical management (25), phenotyping of the remaining majority of IC/BPS patients who lack obvious bladder pathology has been challenging.

In addition, subjects with IC/BPS are at high risk for experiencing other forms of pelvic pain or dysfunction, such as irritable bowel syndrome, which has been attributed to neuronal cross-talk at the level of the spinal cord (26–30). IC/BPS also commonly co-exists with a range of functional somatic syndromes, such as fibromyalgia and myalgic encephalomyelitis, as well as psychiatric comorbidities, such as anxiety and depression. Given the complexity of presentation for many of these patients, phenotyping of patients into more homogeneous groups has proven difficult; no classification system has yet achieved wide-spread use (31–33).

Several previous classification systems, such as UPOINT (34, 35), have focused on optimizing care of patient pain by comprehensively addressing all aspects of their symptomatology. While this can be highly effective at helping to manage individual patient symptoms and improve overall quality of life, the use of such categorization systems requires a significant expertise and familiarity with urologic pain manifestations. More importantly, these systems do not aim to identify the more homogenous groups of patients needed to move forward in mechanistic studies. Thus, the opportunity for cure or prevention of the condition remains elusive for now due to a fundamental lack of understanding of the underlying pathophysiology of bladder pain.

In fact, even the necessity of pain for a diagnosis of IC/BPS is not universally accepted. European Association of Urology (EAU) guidelines consider pain attributed to the bladder a key diagnostic feature (36), while the American Urological Association (AUA) guidelines expand the concept of pain to also include “sensations of pressure or discomfort” (12). The International Association for the Study of Pain (IASP) recently established a new definition for chronic primary bladder pain syndrome which is “chronic pelvic pain perceived in the region of the urinary bladder that is also associated with at least one other symptom,” such as worsening of the pain upon bladder filling and urinary frequency during day time and/or night time. This definition, which is intended to be used in place of IC/BPS, does not require the presence of associated urinary symptoms (37). The guidelines published by the Japanese Urological Association do not require pain for inclusion, stating many patients do not describe pain, instead using the term “hypersensitive” bladder to describe the uncomfortable frequency and nocturia experienced by some individuals without Hunner's lesions.

The differing definitions of IC/BPS have significant consequences in the study of this condition. Our phenotypes reveal that a subset of patients may define their symptoms more as pressure or discomfort than frank pain. This discomfort was more constant and associated with myofascial dysfunction, which was distinct from the perception of pain in the bladder related to voiding cycle. Given the subjective nature of the definitions of IC/BPS, there may be very different populations included if the definition requires a description of “pain” instead of “pain, pressure or discomfort.” As no objective markers of IC/BPS exist, attempting to discover an underlying pathologic mechanism in a pooled, inclusive population of subjects with any form of pressure, pain, or uncomfortable frequency may prevent any potential discoveries.

We focused on identifying the subtle differences in bladder-related symptoms in a population of premenopausal women with a narrowly-defined perception of bladder pain to characterize possible phenotypes that could represent different pathologies. Only by defining more homogeneous populations will we be able to improve therapeutic assignment and outcomes. This has even more significance for future research attempting to discover the underlying molecular mechanisms related to bladder or pelvic pain in each population, a requirement for identifying new approaches to improving patient care. A lack of such homogeneous groups may well be the primary confounding factor affecting studies to date. Only with such knowledge will we be able to define the pathways we can target for better treatment and prevention. While it will be necessary to validate the classification system and optimize the associated phenotypic measures in larger populations, this study is a first step toward those goals.

The clear differences seen in these populations in terms of their responses to treatment support the concept that these phenotypic groups represent distinct, independent disease categories or etiologies. The mechanisms of each of these therapies suggests possible origins of the pain seen in each group, which we hope to evaluate further in additional studies. Most responders to bladder-directed therapies, such as bladder analgesics and intravesical instillations were those with bladder-centric pain related to the voiding cycle categorized to the BPS group. This finding implicates a bladder-derived pathology in these patients, as could be mediated by local inflammation (38, 39) or urothelial dysfunction (40), two mechanisms previously proposed for IC/BPS. The great majority of responders to PFPT were MFP subjects, implicating a myofascial involvement for these individuals (22). The NUPP group did poorly with the common approaches attempted in this population, suggesting that the standard IC/BPS algorithm may be inappropriate for these patients. Given the pattern of pain, this group may represent a vestibulodynia subset with pain referred to the bladder, but for whom treatment of the bladder itself provides little relief.

The myofascial pain group is challenging to recognize without more accurate metrics, because it is common for patients with bladder pain to have some myofascial pain that accompanies their bladder-centric symptoms and frequently will respond to PFPT (41). This is reinforced in the SOM analysis, in which nearly half of the patients with dominant bladder-centric pain also manifest some degree of myofascial pain, albeit less severe than the MFP group. In direct comparison, the two groups exhibited highly similar urinary symptoms, but the BPS group could be clearly distinguished by voiding-cycle related pain, exacerbated by bladder filling. In contrast, the largest factors discriminating the MFP population from BPS were inconsistent levels of defecatory dysfunction, a sensation of incomplete emptying, and small volume incontinence. This mirrors well what has been seen in prior phenotyping studies (31). Each of these individual symptoms, however, were only present in a subset of the overall MFP population. While the two groups were easily recognizable at the population level, it may be difficult to classify patients to one of the two groups on an individual basis using the metrics proposed in this study.

This may be the explanation underlying why the Jaccard coefficient (JC) of the MFP group (0.51) is significantly lower than the BPS group (0.69). A “stable” or “excellent” cluster, in which subjects can be consistently assigned to this group in multiple sub-samplings of the population, will have a Jaccard coefficient of more than 0.70. While the symptoms of these patients were highly similar, the presence of pain with bladder filling was highly specific for the BPS group, which likely aided in the stability of group assignment. In contrast, no single clear symptom was consistently found in all MFP subjects. Regardless of this finding, the difference in treatment responses suggests that the differentiation of the MFP subjects from BPS-dominant groups is clinically relevant and pathologically meaningful. Additional studies with more extensive phenotyping may be helpful in identifying more discriminatory symptomatic features, but we propose that MFP be suspected in IC/BPS subjects without clear pain with bladder filling who complain of POP-like symptoms without objective signs of visceral or pelvic floor descent on exam.

The NUPP group exhibited milder urinary symptoms than the other two groups but was still highly bothered by the pain. The pain was prominent in the distal vagina/urethra and was associated not with bladder filling but with voiding. It is easy to see how these painful symptoms could be attributed to the bladder, despite the distinct pattern in their manifestation. These patients do not respond to the bladder-directed therapies of the BPS group, or indeed any of the other typical IC/BPS therapies documented in this study. A large limitation to understanding the nature of this group was the limited exploration of vaginal and urethral symptoms present in the fGUPI. The questions distinguishing the NUPP group were yes/no questions merely describing pain location or aggravating factors, without a more subtle assessment of these pain manifestations or their severity. These symptoms may have been missed or even overemphasized in some. A more detailed exploration of uncomfortable vaginal and pelvic symptoms in combination with a detailed exam is needed to understand the range of symptoms and the relationship of this phenotype to other pain conditions, such as vestibulodynia.

Interestingly, we did observe the expression of features characteristic of multiple phenotypes by smaller numbers of subjects. This may mean these phenotypes are not sufficiently defined. It has been noted elsewhere that patients often find the language of urology unfamiliar at best and have difficulty describing their symptoms accurately (42), which may limit the utility of patient-reported measures in symptomatic phenotyping. Further, the reticence of subjects to describe their experience fully for a variety of reasons is an additional limiting factor (43). On the other hand, the manifestation of multiple phenotypes within a single patient may simply indicate that these phenotypes accurately describe the different patterns of pain, which are distinct from each other but can co-exist. If occurring independently, individual subjects might express more than one phenotype concurrently.

It was common for the BPS subjects to manifest at least some MFP symptoms. Pelvic floor hypertonicity and tenderness is hypothesized to be a consequence of local pelvic pain and is seen in a variety of pelvic pain conditions (22, 41). These data support this. Interestingly, the converse is not similarly true (e.g., high levels of myofascial pain do not frequent co-occur with mild bladder-centric pain). These data reinforce the concept that myofascial pain is an independent condition, more experienced as “pressure or discomfort” that can be expressed or experienced as the perception of bladder pain in the absence of pain related to voiding cycle.

The group with high levels of all types of pain comprises a small, but not insignificant portion of the overall population. Patients with more domains of pain typically have more severe symptoms (34, 44), suggesting that severe pain in one domain could augment other types of pelvic pain in a positive feedback loop. It is also possible that this group may represent patients with all three phenotypes of pain occurring independently or a more centralized pain, as seen in central sensitization syndrome, who may express more global allodynia. The distance of this phenotype from other patient clusters in the SOM suggests additional or different mechanisms at play in these patients, which may make them more unlikely to respond to locally-directed therapies. Prospective evaluation of this population is critical in understanding whether these subjects represent a separate etiology or more of an end-stage of the other “pure” phenotypes if not effectively treated.

The details of the phenotypic classification determined in this study highlight the challenges in attempting to identify more homogeneous phenotypes of perceived bladder pain, even when narrowly defined by multiple inclusion criteria. It is possible that a larger sample size would identify additional phenotypes that were not recognized here and that are less common or inadequately assessed by the information collected in these questionnaires.

The responder analysis was not anticipated in the original study; thus, the utility of this data is limited by its retrospective acquisition, the absence of more detailed, quantitative outcome measures, and lack of standardized care pathways. In addition, lack of follow up for a subset of the population secondary to reduced clinical access as a result of the SARS-CoV2 pandemic also limited these data. Therefore, while treatment response data are suggestive that the groups respond differently, more detailed prospective studies are needed to validate these preliminary findings.

As this study only included pre-menopausal women, it is not known whether this approach will be generalizable to other age groups or genders. Postmenopausal women can commonly experience a range of genitourinary symptoms associated with that state of relative hormone deficiency, including pain, burning, and dysuria which may complicate this phenotypic classification.

Finally, the IC/BPS subjects included in this analysis did not routinely undergo cystoscopic evaluation as part of their initial treatment course, making it difficult to determine whether a subset of these subjects may have had Hunner's lesions. As Hunner's ulcers are present only in a minority of IC/BPS cases and tend to occur predominantly in older patients, we anticipate that this is a minor portion of this population (45–47). We cannot, however, make any determinations about the influence of Hunner's ulcers on our phenotyping system; the association of individual phenotypes with cystoscopic findings of bladder-specific inflammation or frank ulceration of the urothelium needs to be assessed in future studies. Regardless, as the phenotyping described in this study entirely uses information obtained from patient-reported outcomes at the initial consultation before treatment or examination, such a classification system may still be highly useful, particularly for the non-specialty provider without access to cystoscopic assessment.

Going forward, it will be necessary to validate these phenotypes in larger, multicenter populations. It will be especially important to confirm prospectively that these phenotypes are reliable and accurate predictors of response to treatment. Finally, we must determine if these phenotypes are applicable to other populations such as older women and men. We expect both additional and different symptoms in these groups. Additional studies must determine if new phenotypes are required and how well the model described here can be adapted.

As with all chronic pain conditions, multimodal treatment remains the most effective approach. It is important to address the accompanying extra-pelvic symptoms and recognize the psychosocial and systemic manifestations of this chronic pain condition to improve overall health-related quality of life. We believe, however, that a simple IC/BPS phenotyping into the categories defined here could help focus both initial treatment assignment as well as better facilitate mechanistic studies that could provide insight into disease etiology and promotion. Progress in the care and management of this highly impactful condition is sorely needed; refining our diagnostic categories is a necessary next step in moving toward that goal.

In conclusion, expanded clinical phenotyping of patients reporting perceived bladder pain can identify distinct phenotypes. The different patterns of associated symptoms suggest that these phenotypes may reflect unique pathophysiologies. Further, the varying response of each phenotype to traditional therapies for IC/BPS suggests the utility of such phenotyping and, if validated in prospective studies, may provide a useful approach to understanding the underlying pathophysiologies and improving the diagnosis, treatment, and prevention of bladder pain.

## Data Availability Statement

The datasets generated during and/or analyzed during the current study are available from the corresponding author on reasonable request.

## Ethics Statement

The studies involving human participants were reviewed and approved by Pro00046154. The patients/participants provided their written informed consent to participate in this study.

## Author Contributions

PM assisted in the development of the study, conduct of the research, data analysis, and drafting the manuscript. FK and AC conduct of the research and editing of the manuscript. NJ development of the study, data analysis, and editing of the manuscript. JW, VS, KE, and JTA development of the study, conduct of the research, and editing of the manuscript. DU development of the study, direction of the research, and editing of the manuscript. JEA data management and editing of the manuscript. ALA development of the study, conduct of the research, data analysis, and final approval of the manuscript. All authors contributed to the article and approved the submitted version.

## Funding

This study was funded by a Department of Defense Peer-Reviewed Medical Research Program Discovery Award (PR161914).

## Conflict of Interest

ALA is an expert witness for Cynosure and a consultant for Watershed Medical. KE is a consultant for Allergan and Coloplast. The remaining authors declare that the research was conducted in the absence of any commercial or financial relationships that could be construed as a potential conflict of interest.

## Publisher's Note

All claims expressed in this article are solely those of the authors and do not necessarily represent those of their affiliated organizations, or those of the publisher, the editors and the reviewers. Any product that may be evaluated in this article, or claim that may be made by its manufacturer, is not guaranteed or endorsed by the publisher.
